# Evaluation of self-reported confidence amongst radiology staff in initiating basic life support across hospitals in the Cape Town Metropole West region

**DOI:** 10.4102/sajr.v23i1.1720

**Published:** 2019-11-20

**Authors:** Isak D. Vorster, Steve Beningfield

**Affiliations:** 1 Department of Diagnostic Radiology, Faculty of Health Sciences, University of Cape Town, Cape Town, South Africa; 2 Division of Radiology, Faculty of Health Sciences, University of Cape Town, Cape Town, South Africa

**Keywords:** Cardiac arrest, basic life support (BLS), cardiopulmonary resuscitation (CPR), radiology staff, radiologists, radiology department, confidence levels

## Abstract

**Background:**

The immediate response to cardiac arrest is regarded as the most time-critical intervention. First responders for cardiac arrests in imaging departments are often radiology staff. The study aim was to determine radiology staff members’ confidence in initiating basic life support.

**Objectives:**

The objectives of this study included determining the general confidence levels regarding identifying cardiac arrest and initiation of basic life support (BLS) amongst Radiology staff within the studied sites, as well as to identify potential areas of uncertainty. Another objective included identifying what would contribute to increasing levels of confidence and competence in identifying cardiac arrest and initiating BLS.

**Method:**

A multi-centre cross-sectional survey was conducted using peer-validated, anonymous questionnaires. Questionnaires were distributed to radiology staff working in public sector hospitals within the Cape Town Metropole West. Due to the limited subject pool, a convenience sample was collected. Data were therefore statistically analysed using only summary statistics (mean, standard deviation, proportions, and so on), and detailed comparisons were not made.

**Results:**

We disseminated 200 questionnaires, and 74 were completed (37%). There were no incomplete questionnaires or exclusions from the final sample. Using a 10-point Likert scale, the mean ability to recognise cardiac arrest was 6.45 (SD ± 2.7), securing an airway 4.86 (SD ± 2.9), and providing rescue breaths and initiating cardiac compressions 6.14 (SD ± 2.9). Only two (2.7%) of the participants had completed a basic life support course in the past year; 11 (14.8%) had never completed any basic life support course and 28 (37.8%) had never completed any life support or critical care course. Radiologists, radiology trainees and nurses had the greatest confidence in providing rescue breaths and initiating cardiac compressions from all the groups.

**Conclusion:**

The study demonstrated a substantial lack of confidence in providing basic life support in the participating hospital imaging departments’ staff. The participants indicated that regular training and improved support systems would increase confidence levels and improve skills.

## Introduction

Cardiac arrest is amongst the most serious and life-threatening emergencies in any hospital setting.^1^ Over 80% of cases of sudden death worldwide are because of cardiovascular causes.^2^ Early detection and implementation of resuscitation have been found to optimise a patient’s chance of survival.^3^ The chances of survival following cardiac arrest are greatly improved by promptly and effectively administering cardiopulmonary resuscitation (CPR) as the first-line intervention.^1^

The median survival rate of in-hospital cardiac arrest is higher than out-of-hospital cardiac arrest.^4^ However, the in-hospital survival varies in different areas, and all areas do not contribute to this better survival rate in equal measure.^5^ Although most cardiac arrests will predictably occur in emergency centres (ECs) and high dependency or intensive care units, some do occur in other parts of the hospital. The imaging or radiology department is such an area of the hospital, as it accommodates patients of all degrees of illness for short periods of time while completing investigations, and with increased degree of illness comes a greater risk of cardiac arrest.

While there are no South African studies directly evaluating the performance of basic life support (BLS) by radiology staff, one did assess in-hospital overall outcomes of cardiac arrest.^6^ This study (also conducted in Cape Town) showed that cardiac arrests in the imaging department had the worst outcomes in the hospital.^6^ A further Cape Town study revealed poor knowledge of the use of defibrillators and low confidence in initiating BLS by EC staff.^7^ International studies focusing on nursing staff’s BLS knowledge and training over the last two decades concluded that, in general, both knowledge and training were deficient.^8,9,10,11,12,13^ It is likely that BLS knowledge and training in local imaging departments are inadequate as well.

The aim of this study was to determine whether radiology staff (radiologists, radiographers and both radiologist and radiography trainees, as well as nursing support staff) from public hospitals in the Western Metro of Cape Town, South Africa, felt sufficiently confident to manage BLS. In other words, would they feel comfortable in recognising cardiac arrest, securing an airway, providing rescue breaths and initiating cardiac compressions should the need arise.

## Methods

A survey design was used in this study. Data were collected from radiology staff at five sites across Cape Town over a 3-month period. The sites included two tertiary centres – Groote Schuur Hospital and Red Cross War Memorial Children’s Hospital – and three secondary centres: Mitchells Plain Hospital, New Somerset Hospital and Victoria Hospital. The metropole’s tertiary centres (including Tygerberg Hospital) combined provide 2631 beds to the public healthcare service in Cape Town and manage more than 700 000 outpatients and 120 000 EC contacts annually. The secondary centres provide 1393 beds to the public health care service in Cape Town and manage more than 240 000 outpatients and 165 000 EC contacts annually.^14^ For the 4.5 million people in the Western Cape who access public health care, a total of 60 531 Computed tomography (CT) scans were performed annually in 2013. Just over 29 000 (48%) of these CT scans were performed in the Western Metropole’s hospitals in the region covered in our study.^15^

Participants included radiologists, radiology trainees, radiographers, radiography trainees and nursing support staff working in the imaging departments of the included centres at the time of the survey. A convenience sample of available prospective subjects was collected as it was appreciated that the pool of potential participants was limited. It was also anticipated that staff may be reluctant to contribute to the survey because of unfamiliarity with the subject matter. The sample was captured over a 3-month period from September to November 2017.

The data collection tool was adapted from that used by Maharaj^7^ who tested the same hypothesis in emergency care staff working in a subset of public and private Cape Town ECs.^7^ The survey provides a series of multiple-choice questions, as well as questions rating the confidence to provide or initiate care, using a 10-point scale. Confidence in recognising cardiac arrest, securing an airway, providing rescue breaths and initiating cardiac compressions were the main variables tested this way. Participants were also asked to indicate the areas of BLS where they felt the least confident, and to suggest the interventions that would make them feel more confident. The demographic data of participants (position in department, experience), details of study site, details on BLS training and previous exposure to cardiac arrest were captured. The survey was anonymised, and personal identifying information was not captured.

The survey was disseminated via e-mails using the institutional SurveyMonkey (San Mateo, USA) account of the Division of Emergency Medicine, University of Cape Town. In addition, hard copy surveys were also distributed. These were manually entered into the SurveyMonkey database by the study team.

After completing the data collection, the SurveyMonkey database was downloaded for analysis. The data are presented using flow charts, tables and figures. Also provided is a breakdown of the various participant staff groups who responded with their experience, as well as the proportions of study sites that contributed to the study. Answers to the multiple-choice survey questions are also given and analysed using proportional breakdowns. Confidence levels of participants are presented using the mean and standard deviation. The data were not considered sufficiently powered to cater for any inferential statistics.

### Ethical consideration

Ethical approval for this study was obtained from the Human Research Ethics Committee of the University of Cape Town (668/2017) prior to data collection. Hospital permission was also obtained from the various site CEOs.

## Results

**FIGURE 1:**
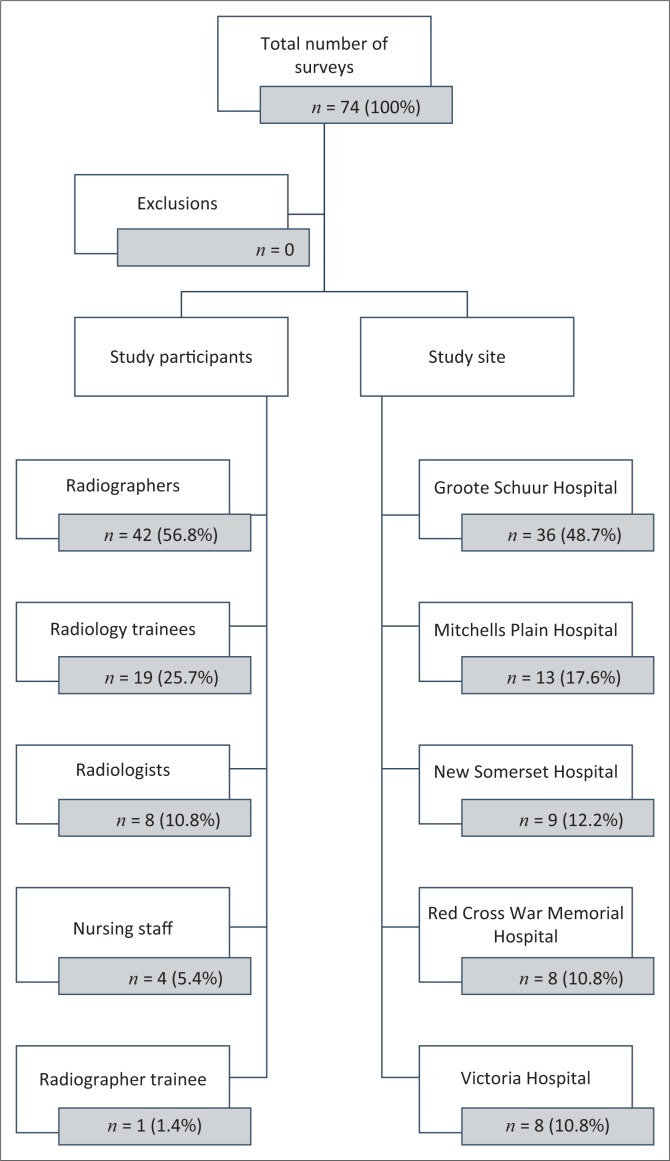
Breakdown of respondents’ demographics.

A total of 200 questionnaires were disseminated via the online survey tool and hand-delivered hard copies. A total of 74 participants responded over the study period giving an overall response rate of 37%. All returned surveys were completed in full and there were no invalid forms. Figure 1 provides a breakdown of the data collection from the various participants and study sites. Of the study participants, 69 (93%) worked exclusively in public sector hospitals and 5 (7%) worked in both public and private sector hospitals.

Of the participants, 35 (47%) had 0–5 years’ radiology experience, 19 (26%) had 5–10 years’ experience and 20 (27%) had more than 10 years’ experience in their respective roles. The different groups’ experience is presented in Table 1.

**TABLE 1:** Level of working experience in the imaging department of various study participant groups.

Experience level	Radiology trainee (*N* = 19)		Radiologist (*N* = 8)		Radiographer trainee (*N* = 1)		Radiographer (*N* = 42)		Nurse (*N* = 4)		Total (*N* = 74)	
	*N*	%	*n*	%	*n*	%	*n*	%	*n*	%	*n*	%
0–5 years	19	100	1	12.5	1	100	12	28.5	2	50.0	35	47.0
5–10 years	0	-	4	50.0	0	-	14	33.3	1	25.0	19	26.0
More than 10 years	0	-	3	37.5	0	-	16	38.1	1	25.0	20	27.0

Of the 74 study participants, 41 (55%) reported that there was no formal BLS training at their place of employment. A total of 63 (85%) participants indicated that they had taken part in a BLS course at some time: two (2.7%) had taken part in a BLS course within the past year and 61 (82.4%) had taken part in a BLS course more than a year ago. The remaining 11 (14.8%) reported never having taken part in a BLS or equivalent course. Of the total 74 responses gathered, 11 (14.9%) respondents reported having completed a trauma course, six (8.1%) reported a critical care course, 22 (29.7%) reported advanced life support course and 28 (37.8%) study participants reported never having completed a trauma, critical care or similar advanced life support course.

A total of 72 (97.0%) reported that they were involved in resuscitating a cardiac arrest between 0 and 5 times per month. No further details were collected on the specifics on these events. No participants reported involvement in more than five cardiac arrests per month other than for two (2.7%) participants, who reported exposure to more than 10 cardiac arrests per month – both reported by radiographers, one based at Red Cross War Memorial and the other based at Groote Schuur Hospital. Table 2 describes the average confidence levels of each group in terms of recognising cardiac arrest, managing the airway and initiating CPR or chest compressions.

**TABLE 2:** Mean (and standard deviation) self-reported confidence levels of participants out of a score of 10 (where 10 was very confident and 0 was no confidence).

Confidence variable	Radiology trainee (*N* = 19)		Radiologist (*N* = 8)		Radiographer trainee (*N* = 1)		Radiographer (*N* = 42)		Nurse (*N* = 4)		Total (*N* = 74)	
	*N*	SD	*n*	SD	*n*	SD	*n*	SD	*n*	SD	*N*	SD
Recognising cardiac arrest	8.95	±1.4	7.75	±1.5	4.0	±0	5.12	±2.3	6.50	±3.4	6.45	±2.7
Managing an airway	7.63	±1.4	4.88	±2.4	3.0	±0	3.48	±2.5	6.75	±3.5	4.86	±2.9
Providing rescue breaths and initiating cardiac compressions	8.74	±0.9	6.38	±1.6	4.0	±0	4.79	±2.9	8.0	±1.2	6.14	±2.9

SD, standard deviation.

**FIGURE 2:**
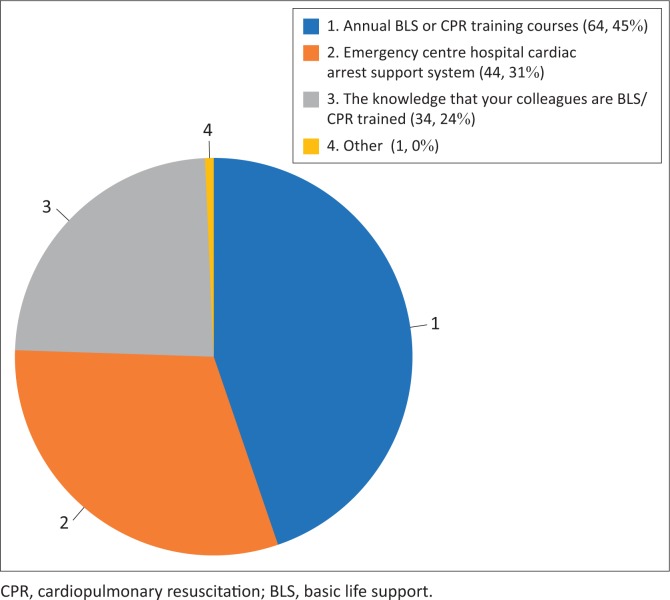
Factors considered to increase confidence of basic life support initiation

Of the 74 study participants, 18 (24%) reported that they were confident in performing all three of the core components of BLS. However, 31 (42%) of participants reported that basic airway management was the component of BLS that they felt least confident with. This was followed by basic circulation support (providing chest compressions) where 26 (35%) of participants reported poor confidence. This was followed by basic breathing assistance and support with 24 (32%) of participants reporting poor confidence. There were 55 (74.32%) participants who stated that they would feel more confident in performing BLS if there was an EC doctor or nurse present. Figure 2 describes the self-reported factors that would increase their confidence in recognising and managing cardiac arrest.

## Discussion

There was a clear deficiency overall in participant confidence for recognising cardiac arrest, managing an airway, providing rescue breaths and initiating cardiac compressions during cardiac arrest. Although radiologists and radiology trainees reported amongst the greatest confidence in recognising cardiac arrest, the confidence levels of radiographers, their trainees and nurses in recognising cardiac arrest, were low. This was concerning, particularly as the latter group is most likely to be amongst first responders in a cardiac arrest. Radiology trainees’ confidence was fairly robust concerning managing an airway, providing rescue breaths and initiating cardiac compressions although nurses reported better confidence in providing rescue breaths and initiating cardiac compressions.

The reasons for these discrepancies were not tested, but may stem from less exposure to actual arrests, as well as prior and current medical training and experience. Radiology trainees are more likely than any of the groups to have been recently exposed to advanced life support training. Low confidence levels in the radiography cohort may result in delays in initiating BLS which may lead to poorer patient outcomes.^16^

Of course, this study only reports confidence levels and not actual performance which can both be better or worse than the reported confidence would suggest. Numerous international studies have shown lack of confidence in initiating BLS when confronted with cardiac arrest, not only amongst general nurses, students or non-surgical disciplines, but also within emergency care staff.^16,17,18,19,20^ Maharaj concluded that emergency care staff working in both public and private settings in South Africa were not sufficiently confident to initiate BLS. As in our study, they showed that the presence of an appropriately trained clinician leading resuscitation boosted confidence in performing BLS.^7^

The lack of regular exposure to cardiac arrest may result either to a degree of complacency or excessive insecurity, leaving staff unprepared to deal with the situation adequately. However, this could potentially be mitigated by ensuring regular BLS refresher courses, together with simulation training.^18^ Only 2.7% of participants in our study had taken part in BLS updates and training over the previous year. This is a far lower proportion than reported in the local study by Maharaj, where 42% of participants indicated that they had participated in a BLS course within the past year and yet they still reported low confidence.^7^ Also locally, Mabasa suggested that regular advanced life support skills training and the implementation of better support systems would contribute greatly to the positive outcomes of cardiac arrest patients.^6^ Still local, Keegan concluded that even nursing staff who attended single BLS training sessions retained limited amounts of knowledge and emphasised that repetitive refresher training is of utmost importance.^10^

Less than half of our study participants were aware of how to obtain BLS training within their setting. Although not specifically determined, the actual availability of training may conceivably be considered a hindrance by removing staff from busy workplaces as well. Maharaj demonstrated the discrepancy between private and public service provision and training, where there was less access to courses in the public sector (which is also where our study took place).^7^ In addition, it is unlikely, considering limited resources, that imaging staff would be prioritised for training.

The option of having the ability to rapidly summon an emergency response team was also popular amongst 34 (45.9%) of our study participants. As has been shown, an emergency response team has a significant positive impact on patient care and in-hospital survival outcomes.^21^ Our study did not collect information regarding the emergency response teams or protocols for any of the sampled institutions. Nonetheless, BLS still needs to be initiated by the first responder irrespective of the clinical setting while awaiting an experienced team to take over life support interventions.

This study provides an interesting perspective on the challenges in achieving a confident team in the radiology department. There were limitations to this study. It included only a proportion of Cape Town hospitals and excluded the private sector. It was also small and the response rate, despite effort from the study team, was lower than anticipated. Data collection was challenging because of the various work patterns and different work areas of staff. It was anticipated that staff may be reluctant to participate, given their expected low confidence levels in managing cardiac arrest.

As such, it was not possible to analyse groups in more detail or compare groups statistically. It was therefore difficult to generalise the findings beyond this sample. Despite the study being limited by its size, the findings echo what is already known elsewhere about the subject. Participant replies were consistent with those from other research. It is believed unlikely that a larger sample would yield a markedly different result.

Although the survey prompted participants to explain their choice whenever ‘Other’ was selected as an answer option, this was not performed by any participant. It is unclear why this was the case but may relate to the survey design.

Because of these limitations, the findings of this study cannot be generalised or offer definitive direction on the BLS confidence of radiology staff in public sector hospitals in Cape Town.

## Conclusion

Although small, this study showed that many radiology staff and their medically trained support staff within a subset of public hospitals in Cape Town, South Africa, felt insufficiently confident in recognising cardiac arrest, managing the airway and initiating cardiac compressions. It further highlighted the lack of uptake or awareness of regular and refresher BLS training within this cohort. It would seem reasonable that advertising and access to BLS training, including use of simulation for imaging staff should be re-evaluated as a patient safety initiative. This would require validation and balancing against the other resources used to manage the burden of acute illness and death within public hospitals. The survey tool used for this study could be used to review the retention of core BLS knowledge and skills post-training interventions. Similarly, opportunities to improve emergency response teams, as well as exposure to cardiac arrest scenarios could be explored in partnership with local ECs, training departments or medical emergency teams. Future exploration by means of a larger study cohort to allow subgroup analysis, specifically considering how prior experience and current training and exposure affect confidence, is recommended.
