# Corrigendum: Evaluation of self-reported confidence amongst radiology staff in initiating basic life support across hospitals in the Cape Town Metropole West region

**DOI:** 10.4102/sajr.v25i1.2269

**Published:** 2021-12-01

**Authors:** Isak D. Vorster, Steve Beningfield

**Affiliations:** 1 Department of Diagnostic Radiology, Faculty of Health Sciences, University of Cape Town, Cape Town, South Africa; 2 Division of Radiology, Faculty of Health Sciences, University of Cape Town, Cape Town, South Africa

In the version of the article initially published, Vorster ID, Beningfield S. Evaluation of self-reported confidence amongst radiology staff in initiating basic life support across hospitals in the Cape Town Metropole West region. S Afr J Rad. 2019;23(1), a1720. https://doi.org/10.4102/sajr.v23i1.1720, the ORCID of the second author was given incorrectly. The correct ORCID should be https://orcid.org/0000-0003-0727-2650 instead of https://orcid.org/0000-0001-7805-7347 in the ‘Authors’ section.

This correction does not alter the study’s findings of significance or overall interpretation of the study’s results. The authors apologise for any inconvenience caused.

